# Second haploidentical bone marrow transplantation with antithymocyte antibody-containing conditioning regimen for graft failure in eight patients with severe aplastic anemia

**DOI:** 10.1038/s41598-024-52917-4

**Published:** 2024-01-27

**Authors:** Chengtao Zhang, Yutong Hou, Yan Yang, Jingjing Zhang, Xiaoli Zheng, Jinsong Yan

**Affiliations:** 1 Department of Hematology, Liaoning Medical Center for Hematopoietic Stem Cell Transplantation, The Second Hospital of Dalian Medical University, No. 467, Zhongshan Road, ShaHeKou District, Dalian, 116027 China; 2 Liaoning Key Laboratory of Hematopoietic Stem Cell Transplantation and Translational Medicine, Blood Stem Cell Transplantation Institute, Dalian Key Laboratory of Hematology, Diamond Bay Institute of Hematology, The Second Hospital of Dalian Medical University, Dalian, 116027 China; 3 Department of Pediatric, Pediatric Oncology and Hematology Center, The Second Hospital of Dalian Medical University, Dalian, 116027 China; 4 Department of Hematology, Air Force Medical Center, PLA, No. 3 Fuchen Road, Haidian District, Beijing, 100142 China

**Keywords:** Health care, Medical research

## Abstract

The effects of a second haploidentical bone marrow transplantation with an antithymocyte antibody-containing conditioning regimen after graft failure in patients with severe aplastic anemia remain unclear. Eight severe aplastic anemia patients with graft failure with a median age of 12.5 (range, 3–22) years were retrospectively reviewed. At the second transplantation, they received a median mononuclear cell number of 15.7 (range, 11.2–20.9) × 10^8^/kg or a median CD34^+^ cell number of 6.2 (range, 2.5–17.5) × 10^6^/kg. They were all successfully engrafted, with a median time of 12.5 (range, 11–16) days for neutrophils and 24 (range, 14–50) days for platelets. Three patients developed skin acute graft-versus-host disease Grades I–II, and another 3 developed limited chronic graft-versus-host disease. All patients successfully recovered after treatment with methylprednisolone (0.5–1 mg/kg/day) and tacrolimus. One patient each died of respiratory failure caused by multidrug-resistant Klebsiella pneumoniae at 8 months and invasive fungal disease at 23 months after transplantation. Six patients survived with a 5-year estimated overall survival of 75% and a median follow-up time of 61 (range, 8–129) months. A second haploidentical bone marrow transplantation with an antithymocyte antibody-containing conditioning regimen was feasible for saving severe aplastic anemia patients with graft failure.

## Introduction

Allogeneic hematopoietic stem cell transplantation (HSCT) has been adopted as one curative option for patients with severe aplastic anemia (SAA)^1–3^, with a long-term overall survival (OS) of 78%–93.9%, irrespective of varied conditioning regimens and donors^4–9^. Due to the insufficiency of siblings or unrelated donors (URDs) with a full human lymphocyte antigen (HLA) match, only approximately 30–35% of patients can promptly find available HLA-matched donors^10^. In contrast, haploidentical donors are easily found; therefore, haploidentical bone marrow transplantation (haplo-BMT) has emerged as a promising treatment with an equivalent effect to that of allogeneic HLA-matched HSCT^11^. Haploidentical BMT with an antithymocyte antibody (ATG)-containing conditioning regimen has been widely employed for treating leukemia, myelodysplasia syndrome, SAA, and more and has achieved promising outcomes, particularly in China^1,9,12^. For instance, a 3-year overall survival (OS) of 84.5 ± 5.0% has been achieved in severe aplastic anemia (SAA) patients with haplo-BMT.

However, graft failure (GF) is a barrier to successful allogeneic BMT; its incidence is approximately 3.8–15% and is higher in nonmalignant hematologic disorders than in hematological malignancies^13,14^. As a lethal complication, it leads to severe bone marrow failure syndrome and a higher risk for death^13^. Hence, patients with GF urgently need to be salvaged with a second allogeneic transplantation.

Unfortunately, patients with GF are very rare, especially patients with SAA, so the effectiveness of a second haploidentical BMT remains limited. Herein, eight SAA patients with GF were retrospectively collected, and the effects of a second haploidentical BMT with ATG-containing conditioning were analyzed. The information on effects may be helpful for future patients with GF.

## Materials and methods

### Patients

Eight SAA patients who underwent a second allogeneic BMT in the Department of Hematology at the Second Hospital of Dalian Medical University (China) and the Air Force Medical Center of PLA (China) from May 2014 to December 2020 were enrolled in this study.

All patients developed GF after the first allogeneic transplantation and had no history of ATG-based immunosuppressive therapy prior to the first allogeneic transplantation.

### Ethical approval

All procedures and treatment protocols were approved by the institutional review boards and the ethics committee in the Second Hospital of Dalian Medical University. The study was in accordance with the Declaration of Helsinki. All patients provided written informed consent.

### Conditioning regimens

In the second haploidentical BMT, the Flu/Cy/ATG conditioning regimen was utilized, which consisted of fludarabine (Flu, 30 mg/m^2^) once a day for 4 consecutive days, cyclophosphamide (Cy, 30 mg/kg) once a day for 4 days, and rabbit ATG (SangStat, France) at 2.5 mg/kg once a day for 2 days (total dose, 5 mg/kg)^12^.

In the first allogeneic transplantation, 4 out of 8 recipients underwent haploidentical BMT with Flu/Bu/Cy/ATG conditioning, as described previously^12^. The remaining 4 recipients underwent HSCT from human leukocyte antigen (HLA)-matched unrelated donors (URDs) with Flu/Cy/ATG conditioning, which consisted of Flu (30 mg/m^2^) once a day for 5 days, Cy (50 mg/kg) once a day for 3 days, and rabbit ATG (2.5 mg/kg) once a day for 4 days^15^. Of note, a total dose of 10 mg/kg ATG was utilized in the first allogeneic transplantation.

### Granulocyte colony-stimulating factor-primed hematopoietic stem cell harvesting

Healthy haploidentical donors were subcutaneously injected with 5 μg/kg granulocyte colony-stimulating factor once a day for 5 consecutive days. Marrow grafts and peripheral blood stem cells were harvested for haploidentical BMT as described previously^12^. Grafts with in vivo T-cell depletion with ATG were employed for two rounds of transplantation.

### Prophylaxis and management for graft-versus-host disease

For two rounds of allogeneic BMT, the same prophylaxis protocol for graft-versus-host disease (GvHD) was used, consisting of tacrolimus, mycophenolate mofetil (MMF), and methotrexate. The detailed protocol was as follows: intravenous tacrolimus (0.03 mg/kg/day) and oral MMF (1.0 g/day) were started on Day 9, and intravenous methotrexate was administered at doses of 15 mg/m^2^ on Day + 1 and 10 mg/m^2^ on Days + 3, + 6, and + 11. The blood concentration of tacrolimus was maintained at 10–20 ng/mL for approximately 3 weeks, and then tacrolimus tablets were used when the recipient’s bowel function recovered to normal. Tacrolimus was gradually tapered at 12 months and discontinued at 15 months after BMT. MMF was tapered at 4 weeks and discontinued at 8 weeks after BMT^12^.

Patients with aGvHD were treated with 1–2 mg/kg/day methylprednisolone, and those who developed steroid-refractory aGvHD were treated with CD25 monoclonal antibody, mesenchymal stem cells, and/or 15 mg of ruxolitinib per day^12^.

### Definitions

Engraftment was defined as the first of 3 consecutive days with an absolute neutrophil count (ANC) greater than 0.5 × 10^9^/L. Primary graft failure was defined as ANC < 0.5 × 10^9^/L by Day + 28, hemoglobin < 80 g/L, and platelets < 20 × 10^9^/L, with donor chimerism < 10%. Secondary graft failure was defined as ANC < 0.5 × 10^9^/L after a successful initial engraftment due to a loss of donor chimerism < 10%^16,17^. aGvHD was diagnosed and graded according to the MAGIC criteria^18^, and cGvHD was diagnosed and graded according to the National Institute of Health Severity score diagnostic criteria (NIH 2014 criteria)^19^. OS was defined as survival from the date of hematopoietic stem cell infusion to death from any cause^12^.

### Statistical analysis

OS is expressed as the median ± standard deviation and was evaluated using the Kaplan–Meier method. All statistical analyses were performed with SPSS version 23.0 (SPSS Inc., USA). *p* < 0.05 was considered statistically significant.

## Results

### Demographic data

As shown in Table 1, at the first allogeneic transplantation, the 8 recipients had a median age of 12.5 (3–22) years old, including 5 males with a median age of 17 (range, 6–22) years old and 3 females with a median age of 7 (range, 3–10) years old. The disease duration from initial diagnosis to the first allogeneic transplantation was 19.5 (1–84) months. Four recipients received haploidentical BMT, and the remaining 4 received allogeneic HSCT from URDs.

**Table 1:** Demographic data and clinical features of eight patients with graft failure after the first allogeneic HSCT.

No	Sex	Age (years)	Diagnosis	Disease duration (months)	Donor/age (years)	Blood typing donor/recipient	HLA matching	MNC (× 10^8^/kg)	CD34^+^ (× 10^6^/kg)	Graft failure	Time to graft failure (days)
#1	M	19	SAA	40	Father/42	AB/AB	5/10	8.2	3.8	Primary	28
#2	M	22	SAA	1	Father/45	AB/B	5/10	8.1	6.6	Secondary	198
#3	F	7	SAA	60	Mother/38	O/O	6/10	12.9	5.2	Primary	22
#4	M	15	SAA	12	Sister/32	A/A	5/10	7.5	4.3	Primary	18
#5	F	10	SAA	15	URD/22	O/A	10/10	11.1	2.1	Secondary	60
#6	F	3	SAA	12	URD/30	O/B	10/10	14.7	12.6	Secondary	90
#7	M	17	SAA	84	URD/29	B/O	10/10	10.1	6.4	Secondary	62
#8	M	6	SAA	24	URD/41	O/A	10/10	7.5	6.3	Secondary	183

*HSCT* hematopoietic stem cell transplantation, *F* female, *M* male, *yrs* years old, *mons* months, *SAA* severe aplastic anemia, *URD* unrelated HLA-matched donor, *HLA* human leukocyte antigen, *MNCs* mononucleated cells.

Before the first transplantation, the eight patients had severe hypoplastic myelopoiesis, and all required blood transfusion and platelet transfusion support. They presented no liver or kidney dysfunction, no treatment with ATG, and no donor-specific anti-HLA antibodies. Mononucleated cells and CD34 + stem cells were infused at 9.15 (7.5–14.7) × 10^8^/kg of recipient body weight and 5.75 (2.1–12.6) × 10^6^/kg of recipient body weight, respectively.

After the first transplantation, 3 out of 8 recipients developed primary graft failure, and 5 developed secondary GF with a median time of 90 (60–198) days. In detail, 4 recipients who received Flu/Cy/ATG conditioning developed secondary GF at 60, 90, 62, and 183 days. They achieved complete chimerism of donor cells but then rapidly lost the donor-cell chimerism; of note, 3 recipients with URDs developed secondary GF at 60–90 days post-transplantation.

Prior to the second transplantation, the 8 patients had a median white blood cell count of 0.23 × 10^9^/L (range, 0.06–1.12 × 10^9^/L), a median neutrophil count of 0.02 × 10^9^/L (range, 0–0.45 × 10^9^/L), a median hemoglobin concentration of 67 g/L (range, 51–96 g/L), and a median platelet count of 19 × 10^9^/L (range, 5–26 × 10^9^/L). The second transplantation was performed at a median interval of 97.5 days (range 28–331) from the first transplantation. Different haploidentical donors were used in six patients, and the same haploidentical donors were used in 2 patients (Table 1). Eight patients received marrow grafts and HBSC grafts with median mononuclear cells of 15.7 (range, 11.2–20.9) × 10^8^/kg and median CD34^+^ cells of 6.2 (range, 2.5–17.5) × 10^6^/kg. They were all successfully engrafted with a median time for neutrophils and platelets of 12.5 (range, 11–16) and 24 (range, 14–50) days, respectively.

### Liver function and renal function

Liver transaminases, such as aspartate transaminase (AST) and alanine aminotransferase (ALT), are useful biomarkers of liver injury in patients with some degree of intact liver function. During the first conditioning, one patient experienced liver dysfunction with mildly elevated enzyme concentrations. Two patients showed an increase in creatinine concentration: one patient's concentration increased to 98.5 µM/L on Day + 99, and the other patient's concentration increased to 111 µM/L on Day + 140. A reduction in the FK 506 dose resulted in a return to normal creatinine concentrations after 1 week.

After the second BMT, only one patient experienced liver dysfunction with mildly elevated enzyme concentrations, and none had renal impairment.

### Infection

During the first allogeneic transplantation, 5 patients experienced infections, 2 of which were caused by multidrug-resistant Klebsiella pneumoniae confirmed by bacterial culture of peripheral blood samples. The two patients recovered after treatment with tigecycline to eliminate Klebsiella pneumoniae sepsis. One patient experienced a fungal lung infection, which was controlled with liposomal amphotericin B. Two patients separately experienced an oral infection and acute throat inflammation, and they recovered after treatment with appropriate antibiotics.

During the second transplantation, 3 of the 8 patients experienced infections. One patient developed sepsis due to Escherichia coli confirmed by bacterial culture of peripheral blood samples, and the patient recovered after treatment with imipenem and cilastatin sodium; 1 experienced perianal soft tissue infection, and recovered after treatment with imipenem-cilastatin sodium and vancomycin; and the remaining 1 suffered from severe pneumonia and recovered after intravenous application of imipenem-cilastatin sodium and voriconazole. Unfortunately, 2 patients died of respiratory failure, one caused by multidrug-resistant Klebsiella pneumonia at 8 months and the other by invasive fungal disease at 23 months after transplantation.

### Viral reactivation and virus monitoring

Before the first HSCT/BMT, one recipient was seropositive for EBV, and one recipient was seropositive for CMV. After the first allogeneic transplantation, EBV was reactivated in three recipients at a median of 28 days (range, 17–30 days), with median EBV loads of 5.0 × 10^3^ copies/ml (range, 2.7 × 10^3^–6 × 10^5^ copies/ml). CMV was reactivated in four recipients at a median of 29 days (range, 20–49 days), with median CMV loads of 6.8 × 10^4^ copies/ml (range, 9.5 × 10^3^–1.0 × 10^6^ copies/ml).

After the second BMT, EBV was reactivated in 3 recipients with median EBV loads of 2.85 × 10^5^ (range, 2.5 × 10^3^- 2.9 × 10^5^) copies/ml at a median of 25 (range, 19–119) days. CMV was reactivated in 4 recipients with median CMV loads of 9.7 × 10^4^ (range, 8.0 × 10^3^–1.5 × 10^6^) copies/ml at a median of 26 (range, 19–35) days. EBV and CMV infections were well controlled with human immunoglobulin, ganciclovir, and/or foscarnet sodium in both allogeneic transplantations.

### Hemorrhagic cystitis

After the first HSCT/BMT, no patient experienced hemorrhagic cystitis (HC). After the second transplantation, 2 recipients experienced hemorrhagic cystitis. In detail, one patient had Grade I HC on Day + 19, concomitant with a BKV load of 1.05 × 10^8^ copies/ml, and another patient had Grade II HC on Day + 17, concomitant with a BKV load of 6.9 × 10^10^ copies/ml. Both patients recovered with antiviral agents.

### aGVHD and cGVHD

After the first HSCT/BMT, one patient experienced skin aGVHD (Grade I), which was successfully treated by the addition of methylprednisolone (0.5 mg/kg/day) for 3 days.

After the second haplo-BMT, 3 (37.5%) patients experienced aGVHD, with skin aGVHD of Grades I-II, which were successfully treated with methylprednisolone (1 mg/kg/day) and tacrolimus. During follow-up post-transplantation, three (37.5%) patients experienced limited skin cGVHD without extensive cGVHD.

### Immune reconstitution

After the second transplantation, the median absolute lymphocyte count reached 130 cells/μL on Day 30 (ALC-30), 873 cells/μL on Day 90, and 1,172 cells/μL on Day 360. CD3^+^ CD4^+^ and CD3^+^ CD8^+^ median T lymphocyte counts were 32 cells/μL and 112 cells/μL on Day 30, 185 cells/μL and 595 cells/μL on Day 90, and 234 cells/μL and 817 cells/μL on Day 360, respectively. B lymphocyte (CD19^+^) median counts were 3 cells/μL on Day 30, 30 cells/μL on Day 90, and 126 cells/μL on Day 360. The median IgA, IgG, and IgM levels were 0.31 g/L, 10.00 g/L, and 1.07 g/L on Day 30; 0.26 g/L, 9.02 g/L, and 0.19 g/L on Day 90; and 0.56 g/L, 8.95 g/L, and 0.55 g/L on Day 360, respectively (Table 2).

**Table 2:** Patient clinical characteristics at second BMT.

No	Days from 1st to 2nd HSCT	Donor/age (years)	HLA matching	Blood typing donor/recipient	MNC (× 10^8^/kg)	CD34 + (× 10^6^/kg)	Time to neutrophil engraftment (days)	Time to platelet engraftment (days)	Survival (months)	Outcome
#1	48	Same donor	5/10	AB/AB	18.0	5.7	14	33	72	Alive
#2	223	Mother/46	5/10	0/AB	16.0	6.8	11	23	46	Alive
#3	40	Father/35	5/10	B/O	20.9	17.5	12	14	52	Alive
#4	28	Same donor/32	5/10	A/A	15.5	16.7	16	20	8	Death
#5	105	Father/32	5/10	A/A	11.2	3.8	15	19	23	Death
#6	116	Father/29	6/10	AB/B	13.5	2.5	13	50	129	Alive
#7	90	Father/45	5/10	O/O	16.1	4.4	12	34	73	Alive
#8	331	Father/35	5/10	A/A	12.8	6.7	12	25	70	Alive

*F* female, *M* male, *HLA* human leukocyte antigen, *MNCs* mononuclear cells, *BM* bone marrow.

### Overall survival and disease-free survival

**Figure 1:**
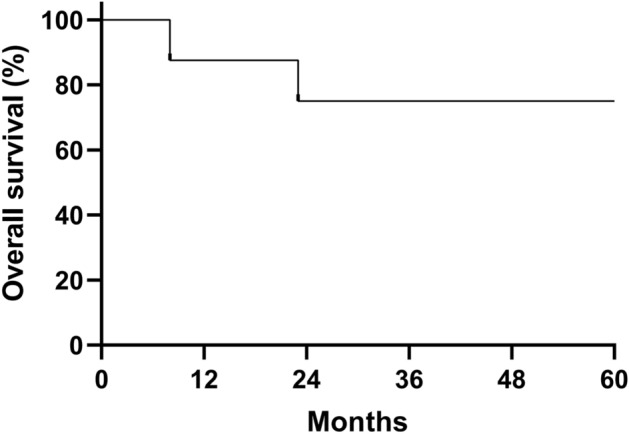
Overall survival (OS) of 8 patients after a second haploidentical bone marrow transplantation. The estimated OS at 60 months was 75%.

Eight patients were followed up through January 31, 2023, with a median follow-up time of 61 (range, 8–129) months. Six survived and 2 died of severe pneumonia at 8 and 23 months, respectively. The five-year estimated OS was 75% using Kaplan‒Meier analysis (Fig. 1).

## Discussion

Life-threatening complications of GF in patients with SAA usually include persistently severe cytopenia, lethal infections, poor performance status, and organ damage. In these situations, haploidentical BMT is an urgent option that can rescue SAA patients with GF^1,20^.

Risk factors for GF are heterogeneous, including HLA disparity between recipient and donor, classification of diseases, numbers of mononucleated cells and CD34 + cells, stem cell sources, varied conditioning regimens, and others. The complex causes usually involve one or more risk factors. After the first transplantation, 8 patients had sufficient CD34 numbers and undetectable DSA antibodies. As described elsewhere, a threefold higher incidence of GF occurs in patients with nonmalignant than with malignant hematologic malignancies^13^. SAA is a nonmalignant hematopoietic disorder, and GF is mainly due to insufficient conditioning and resultant immunosuppression, which preserves residual host T lymphocytes and then causes immune-mediated damage to donor stem cells. Likewise, residual host natural killer cells also result in graft failure by causing graft rejection. In this study, 3 patients who received allo-HSCT from URDs presented with secondary GF at 60–90 days, and they all utilized conditioning consisting of ATG, fludarabine and cyclophosphamide; however, no TBI was added. Conditioning regimens including busulfan and Cy or Flu and Cy are all associated with a higher risk for graft rejection in SAA; however, the addition of low-dose TBI was able to overcome graft failure^21^. Therefore, FLU-CY-ATG conditioning combined with low-dose TBI may have attenuated the rate of graft failure in the 3 patients who developed secondary GF at 60–90 days post-transplantation.

As a nonmalignant disorder, SAA has a higher rate of GF than hematological malignancies, and the total rate of primary GF, secondary GF, and poor graft function rose to nearly 20% in SAA patients when posttransplantation cyclophosphamide (PT/Cy) was used^14^. In contrast, conditioning regimens containing fludarabine and ATG enabled enhanced immunosuppression, facilitated successful engraftment and finally reduced the GF rate in SAA^9,12^. Although total body irradiation (TBI) as part of the conditioning regimen is also beneficial to successful engraftment, it has long-term side effects in some pediatric patients, such as cataracts and secondary malignancies^22^. Therefore, Flu/CY/ATG conditioning with no TBI was employed, and no occurrence of GF after the second transplantation indicated that nonmyeloablative and half-dose ATG were sufficient to ensure successful engraftment after GF in the first transplantation in SAA patients. During follow-up, patients underwent a second transplantation and grew up healthy with no mental retardation.

In hematological malignancies, a second haploidentical BMT with reduced-intensity Flu/Cy conditioning or the Baltimore conditioning protocol (PT/Cy) yielded a one-year OS of 56.6% to 66%, with a relapse rate of 23.1%^23,24^. However, as a nonmalignant disorder, second transplantation for GF in SAA had a much lower risk of disease relapse; for instance, PT/Cy-based haploidentical transplantation achieved successful engraftment, with a 2-year OS of 91.6% for graft failure in patients with nonmalignant disorders^25^. In this study, a second haploidentical BMT with ATG-containing conditioning achieved a 5-year OS of 75% for SAA patients with GF, indicating that a second haploidentical BMT is a rescue option for urgent cases of graft failure in patients with SAA, with either PT/CY-based or ATG-containing conditioning regimens. Additionally, successful engraftment in the 2 patients who used the same haploidentical donors for both transplantations indicated that using unchanged donors is possible when there are no alternative donors^26^; furthermore, it suggested that insufficient immunosuppression of recipients’ T lymphocytes and natural killer cells may be the leading cause of GF in the first transplantation.

Prior to the first allogeneic stem cell transplantation, no chemotherapies was usually administered into patients with nonmalignant hematological disorders, it resulted in no occurrence of immunosuppression, particularly an inhibitory impact on T-cell function, therefore, the GF incidence was higher than in patients with leukemia or high-risk myelodysplasia syndrome. As fludarabine, cyclophosphamide, busulfan, and ATG all had eliminating effects on immunology cells including T lymphocytes, B lymphocytes, and natural killers, etc., then the first conditioning had strongly suppressed the immunological function in patients with GF, as a result, we deduced that a successful engraftment should be ensured even with a reduced dosages of conditioning regimen consisting of fludarabine, cyclophosphamide, and ATG in the second transplantation, additionally, a reduced dosage of conditioning regimen also presented with tolerable toxicities in the second haploidentical BMT, therefore, Flu/Cy/ATG conditioning regimen described previously was employed for the second haploidentical BMT^12^, in which it has successfully saved one SAA patient with GF, suggesting a reduced dosage of Flu/Cy/ATG conditioning may ensure a successful engraftment in the second haploidentical BMT, however, this conditioning regimen may be utilized only at the scenario that ATG-containing conditioning regimen had been used in the first allogeneic stem cell transplantation. Collectively, it is noteworthy that 8 patients with GF in this investigation all had experienced successful engraftments with Flu/Cy/ATG, but the effectiveness of Flu/Cy/ATG for the second haploid-BMT should be further validated by a clinical trial. Additionally, hematopoietic tissues and cells in the marrow microenvironment were usually damaged in SAA prior to the first transplantation and were further severely injured by myeloablative conditioning for the first allogeneic transplantation. Therefore, mesenchymal stem cells were utilized to repair the injured hematopoietic microenvironment, partly restore hematopoiesis and promote engraftment; therefore, MSC-containing marrow grafts combined with PBSC grafts may help to improve engraftment in the second transplantation after GF in the first transplantation.

In the second transplantation, younger patients had better survival due to their tolerance of the conditioning and long-term neutropenia^27^. Eight young patients with a median age of 12.5 years achieved a 5-year OS of 75%, which might have partly benefited from their young ages; hence, second transplantation should be encouraged, particularly for GF in pediatric patients with SAA.

Concomitant infections during the neutropenic period were conducive to a poorer performance status, which was one of the most significant prognostic factors for a worse outcome^27^. In this study, infections were cured during the process of second transplantation; however, 2 patients died after transplantation. Therefore, prevention or intense therapy for infections should be emphasized to reduce infection-related death and improve OS post-transplantation.

To date, second haploidentical BMT with an ATG-containing conditioning regimen for SAA patients with GF has been very rare, and much limited information is available. In this study, the sample size of only 8 patients limited the accuracy of the information; however, a 5-year OS of 75% indicated that second haploidentical BMT with Flu/Cy/ATG conditioning is a feasible and prompt way to salvage SAA patients with GF.

## Data Availability

The original contributions presented in this study are included in the article, and further inquiries can be directed to the corresponding author(s).
